# Zirconium-89-based PSMA PET/CT with delayed imaging for early detection of biochemical recurrence in prostate cancer patients with PSA ≤ 0.2 ng/mL: A feasibility study

**DOI:** 10.1007/s00259-026-07854-x

**Published:** 2026-04-06

**Authors:** Tilman Speicher, Simon Latell, Arne Blickle, Moritz B. Bastian, Stephan Maus, Andrea Schaefer-Schuler, Mark Bartholomä, Muammer Misirci, Caroline Burgard, Samer Ezziddin, Florian Rosar

**Affiliations:** Department of Nuclear Medicine, Saarland University – Medical Center, Kirrberger Str. 100, Geb. 50, D-66421, Homburg, Germany

**Keywords:** PSMA, PET/CT, Zr-89, Zirconium-89, Biochemical recurrence, Low PSA

## Abstract

**Purpose:**

PSMA PET/CT using PSMA ligands labeled with the long-lived radionuclide zirconium-89 represents a promising novel imaging approach for prostate cancer. Preliminary evidence indicates that [^89^Zr]Zr-PSMA PET/CT may enhance the detection of tumor lesions in patients with biochemical recurrence (BCR). This study aims to evaluate the positivity rate and feasibility of [^89^Zr]Zr-PSMA imaging in patients with very low PSA levels (≤ 0.2 ng/mL).

**Methods:**

Sixty-five patients with BCR and PSA levels ≤ 0.2 ng/mL undergoing [^89^Zr]Zr-PSMA PET/CT were analyzed. The mean PSA level at imaging was 0.14 ± 0.04 ng/mL. PET/CT was performed using a mean injected activity of 129.0 ± 19.3 MBq and an uptake time of 48 h. Positivity rates and potential predictors of scan positivity, including PSA level, PSA doubling time, and initial Gleason score, were evaluated.

**Results:**

[^89^Zr]Zr-PSMA PET/CT achieved a high overall positivity rate of 72.3%, with 47 of 65 patients presenting with at least one suspicious lesion. Local recurrence was detected in 25 patients, while lymph node and bone metastases were observed in 29 and 4 patients, respectively. Patients with a Gleason score ≥ 8 exhibited a higher positivity rate than those with lower scores (*p* = 0.047).

**Conclusion:**

In patients with BCR and PSA levels ≤ 0.2 ng/mL, [^89^Zr]Zr-PSMA PET/CT yielded a 72.3% positivity rate in this single-center cohort of 65 individuals. This notable sensitivity at minimal PSA concentrations may enable early identification of recurrent disease and facilitate individualized targeted therapy.

## Introduction

Prostate-specific membrane antigen (PSMA) has been established as a key target for diagnostics and therapy [1–3] in prostate cancer, which is one of the most common malignancies in men worldwide [4]. The PSMA molecule is overexpressed on prostate cancer cells and thus serves as a semi specific target [5–7]. PSMA-targeted PET/CT using various tracers has become established in diagnostics, particularly for primary staging and for the localization of biochemical recurrence (BCR) [8–11]. It is well known that the positivity rate of prostate cancer with [^68^Ga]Ga-PSMA-11 PET/CT increases with rising prostate-specific antigen (PSA) serum levels [12–15]. Especially at very low PSA levels ≤ 0.2 ng/mL, a recently published multicenter study reported that the positivity rate is 29.6% when using standard PSMA tracers with short-lived radionuclides [16]. A possible approach to improve this positivity rate is the use of the long-lived tracer [^89^Zr]Zr-PSMA-617 for PET/CT imaging [17, 18]. ^89^Zr with a half-life of 78.4 h is a positron emitter that is well suited for processes requiring extended imaging times or benefiting from prolonged observation [19–22]. Optimal PET/CT lesion visualization at low PSA levels may require a longer ligand internalization time than is currently achievable with established PSMA tracers [23]. Importantly, long-lived radionuclides permit extended clearance times from normal tissues, thereby improving lesional contrast, which presents a considerable advantage over short-lived tracers [23–27]. Recent studies presenting first clinical experiences with [^89^Zr]Zr-PSMA PET/CT have shown that lesion detection is successful even when standard PSMA PET/CT fails to localize BCR, including at low PSA levels [25–27].

The purpose of this study is to examine the positivity rate and feasibility of [^89^Zr]Zr-PSMA imaging in patients with very low PSA levels ≤ 0.2 ng/mL, which reflect early BCR and carry substantial clinical importance.

## Methods

### Patients and ethics

A total of *n* = 65 consecutive patients with BCR and PSA levels ≤ 0.2 ng/mL undergoing [^89^Zr]Zr-PSMA PET/CT were identified from clinical databases and enrolled in this analysis. Patients underwent [^89^Zr]Zr-PSMA PET/CT imaging between October 2021 and December 2025. Referrals originated from both hospital-based and private practice urologists. The mean age of patients was 67.4 ± 7.3 years, ranging from 53 to 83 years. The mean PSA level at the time of imaging was 0.14 ± 0.04 ng/mL, with values ranging from 0.02 to 0.2 ng/mL. The vast majority of patients underwent radical prostatectomy for primary treatment. The initial Gleason score of the cohort ranged from 6 to 9. Detailed patient characteristics are summarized in Table 1. The study was conducted in accordance with the Declaration of Helsinki and approved by the Institutional Review Board of the Ärztekammer des Saarlandes/Saarbrücken (approval number: 170/22, date of approval: 13th September 2022). All patients provided written informed consent for [^89^Zr]Zr-PSMA PET/CT and consented to the publication of anonymized data.

**Table 1:** Patient characteristics

Patient characteristic	Value
Age (*n* = 65)	
Median	67
Range	53–83
Mean ± SD	67.4 ± 7.3
PSA (*n* = 65)	
Median	0.14
Range	0.02–0.2
Mean ± SD	0.14 ± 0.04
Category, ng/mL, % (n)	
<0.1	13.8% (9)
0.1–0.15	47.6% (31)
>0.15–0.2	38.5% (25)
PSA doubling time, months (*n* = 39)	
Median	5
Range	1–30
Category, months, % (n)	
<3	12.8% (5)
3–6	51.3% (20)
>6–<12	20.5% (8)
≥12	15.3% (6)
Initial Gleason score (*n* = 62)	
Range	6–9
6	4.8% (3)
7	66.1% (41)
8	6.4% (4)
9	22.6% (14)
Primary treatments (*n* = 65)	
Radical prostatectomy	98.5% (64)
Primary radiation therapy	1.5% (1)
Salvage radiation therapy	18.5% (12)
Androgen deprivation therapy	13.8% (9)

### PET/CT imaging

[^89^Zr]Zr-PSMA PET/CT was performed using a median injected activity of 129.0 ± 19.3 MBq (range: 88–173 MBq) and an uptake time of 48 h. Imaging was performed on a Biograph mCT 40 scanner (Siemens Medical Solutions, Knoxville, TN, USA), covering the body area from the vertex to the mid-femur. PET acquisition time was 5 min/bed position. CT scans were acquired in low-dose technique without diagnostic CT and without intravenous contrast administration, using an X-ray tube voltage of 120 keV and automated tube current modulation (CARE Dose4D, Siemens, Erlangen, Germany; maximum tube current 30 mA). Image acquisition was followed by reconstruction with a soft-tissue kernel (B31f) at a slice thickness of 5 mm. PET emission data were corrected for scatter, random coincidences, and radioactive decay. PET reconstruction was performed using an OSEM 3D algorithm with 3 iterations and 24 subsets.

### PET/CT image analysis

Imaging and interpretation were performed as part of clinical routine. [^89^Zr]Zr-PSMA PET/CT findings were classified visually by consensus of at least two nuclear medicine specialists. As image interpretation was conducted in routine clinical practice, relevant medical history and prior imaging findings were taken into account when available. Scans were classified as positive if clearly discernible tracer uptake was observed on 48-hour PET imaging in locations typical for recurrence or metastatic disease. Scans were considered negative if no pathological foci were detected. For quantitative analysis in this study, tracer uptake on [^89^Zr]Zr-PSMA PET/CT, the maximum standardized uptake value (SUV_max_) of each suspicious lesion was measured using SyngoVia Enterprise VB 60 software (Siemens Healthineers, Erlangen, Germany).

Imaging findings were substantiated and validated through longitudinal follow-up, including subsequent assessment of serum PSA levels after treatment, determining (best) PSA response. Follow-up data were collected and analyzed until February 2026.

### Endpoint and statistical analysis

Scan positivity, defined as the identification of at least one suspicious lesion, served as the primary endpoint, allowing calculation of the overall positivity rate, defined as the proportion of patients with a positive scan relative to the total cohort. All descriptive statistics were performed using PRISM 10 (GraphPad Software, San Diego, USA). The influence of different parameters (PSA level, PSA doubling time, and Gleason score) on the positivity rate was investigated. PSA doubling time (PSA-DT) was calculated based on an exponential growth model from prior PSA levels. A *p*-value < 0.05 was defined as the threshold for statistical significance. Chi-square test and ANOVA were performed to assess statistical significance.

## Results

**Fig. 1:**
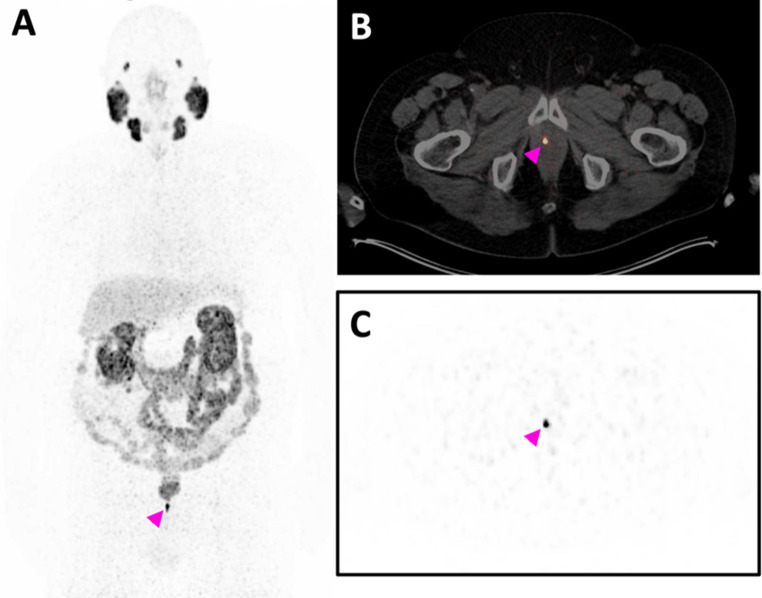
Patient example of [^89^Zr]Zr-PSMA PET/CT demonstrating a local recurrence. (**A**) Maximum intensity projection (MIP), (**B**) CT fused with PET data, (**C**) PET data

**Fig. 2:**
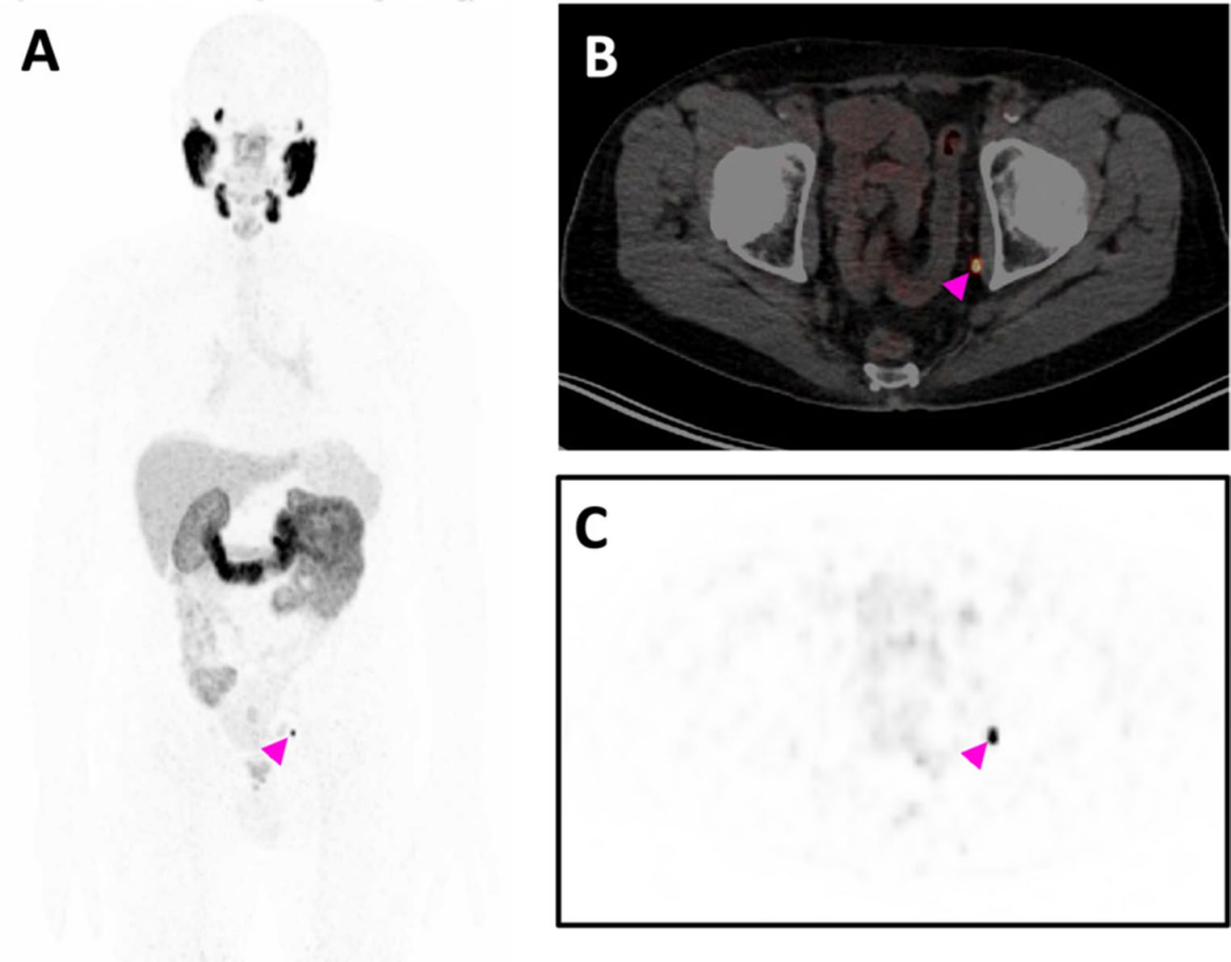
Patient example of [^89^Zr]Zr-PSMA PET/CT showing a left-sided lymph node metastasis adjacent to the left obturator internus muscle. (**A**) Maximum intensity projection (MIP), (**B**) CT fused with PET data, (**C**) PET data

In this cohort of 65 patients with PSA levels ≤ 0.2 ng/mL, 47 patients presented with at least one suspicious lesion on [^89^Zr]Zr-PSMA PET/CT, yielding an overall positivity rate of 72.3% (47/65 patients). Positive findings were observed across various patterns of disease involvement. Lesions were identified at both local and metastatic sites. Local recurrence was detected in 25 patients, while lymph node and bone metastases were observed in 29 and 4 patients, respectively. No visceral metastases were observed. Among patients with positive findings, most tumor manifestations were unifocal (24/47, 51.1%). Smaller proportions showed bifocal (12/47, 25.5%), oligofocal (≤ 5 lesions; 8/47, 17.0%), or multifocal disease (> 5 lesions; 3/47, 6.4%). Representative examples of positive [^89^Zr]Zr-PSMA PET/CT scans are shown in Figs. 1 and 2, demonstrating successful localization of BCR in form of local recurrence (Fig. 1) and lymph node metastases (Fig. 2), respectively.

**Fig. 3:**
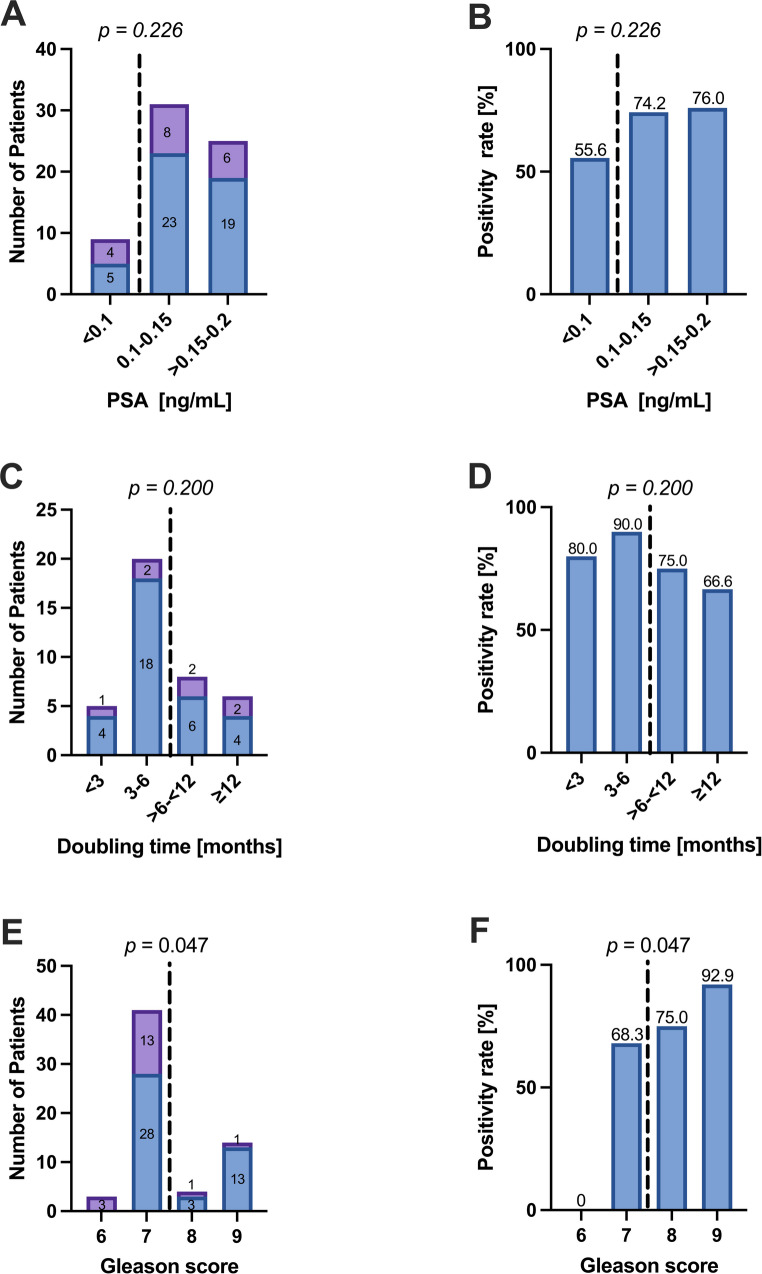
Proportion of patients with positive/negative scans and positivity rate stratified by (**A/B**) PSA levels, (**C/D**) PSA doubling time (PSA-DT) and (**E/F**) Gleason score

The proportions of positive/negative scans and the corresponding positivity rates separated by different levels of serum PSA are depicted in Fig. 3A**/B.** For PSA levels < 0.1 ng/mL a positivity rate of 55.6% (5/9 patients) was recorded. In case of slightly higher PSA levels between 0.1 and 0.15 ng/mL, the positivity rate was 74.2% (23/31 patients). For PSA levels > 0.15 ng/mL it was 76.0% (19/25 patients). Although analysis showed no statistically significant difference *(p = 0.226)*, we observed a lower positivity rate in patients with < 0.1 ng/mL (55.6%), compared to patients with higher PSA levels (75.0% positivity rate), indicating a discernible trend toward reduced detectability at very low PSA concentrations.

PSA-DT was available for 39 of 65 patients (60.0%). The proportions of positive/negative scans and the corresponding positivity rates separated by different PSA-DT are depicted in Fig. 3C**/D**. Positivity rates were 80.0% (4/5 patients) for < 3 months, 90.0% (18/20 patients) for 3–6 months, 75.0% (6/8 patients) for > 6 - <12 months, and 66.6% (4/6 patients) for ≥ 12 months. No statistically significant difference was observed at a 6-month cutoff (*p* = 0.200). Nevertheless, observed scan positivity was lower in patients with PSA-DT longer than 6 months (71.4 vs. 88.0%), pointing toward a trend of diminished detectability.

Data on the initial Gleason score were available for 62 of 65 patients (95.4%). The proportions of positive/negative scans and the corresponding positivity rates separated by different Gleason score are depicted in Fig. 3E**/F**. Positivity rates were 68.3% (28/41 patients) for those with a Gleason score of 7, 75.0% (3/4 patients) for a score of 8, and 92.9% (13/14 patients) for a score of 9. In none of the three patients with Gleason score 6 tumor lesions could be identified. A statistically significant difference was observed at a threshold of Gleason score 8, with higher positivity rate for patients with a score equaling or higher than this cut-off (88.9% vs. 63.6%; *p* = 0.047). The association between binarized clinical parameters and the positivity rate of [^89^Zr]Zr-PSMA PET/CT is presented in Table 2.

**Table 2:** Association between binarized clinical parameters and the positivity rate of [^89^Zr]Zr-PSMA PET/CT

Category	Threshold	Positivity rate [%]	*p*-value	phi-coefficient
PSA	< 0.1	55.6	0.226	0.150
	≥ 0.1	75.0		
PSA-DT	≤ 6	88.0	0.200	−0.207
	> 6	71.4		
Gleason score	< 8	63.6	0.047	0.253
	≥ 8	88.9		

**Fig. 4:**
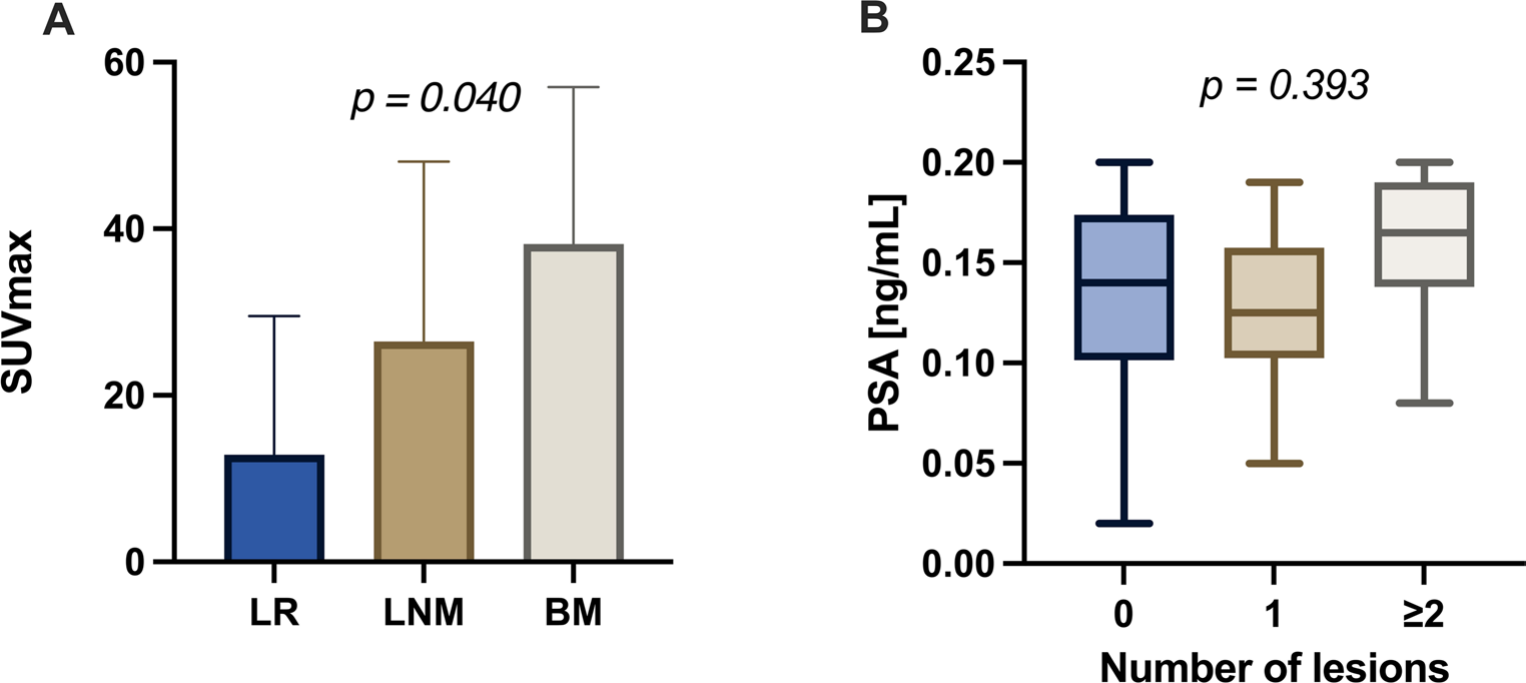
(**A**) SUV_max_ in [^89^Zr]Zr-PSMA PET/CT for local recurrence (LR), lymph node metastases (LNM), and bone metastases (BM). (**B**) PSA levels according to the number of tumor lesions detected

The overall mean of the measured SUV_max_ values across all lesions was 19.2 ± 18.3. Figure 4A illustrates SUV_max_ values stratified by tumor lesion category, including local recurrence (LR), lymph node metastases (LNM), and bone metastases (BM). Mean SUV_max_ was lowest in lesions classified as local recurrence (12.9 ± 16.7), intermediate in lymph node metastases (26.4 ± 21.6), and highest in bone metastases (38.2 ± 18.8). Differences in SUV_max_ among lesion categories were statistically significant (*p* = 0.040), indicating increasing tracer uptake from local recurrence to metastatic lesions. Figure 4B depicts PSA levels stratified by the number of detected tumor lesions, with no statistically significant association observed (*p* = 0.393).

Follow-up data, including information on subsequent therapy and PSA kinetics, were available in 42 of 47 patients (89.4%) with positive findings. Among these patients, 30 (71.4%) received targeted radiotherapy guided by [^89^Zr]Zr-PSMA PET/CT. In patients who underwent PET-guided radiotherapy, the mean decline in PSA levels following treatment was 70.4 ± 31.6%. A PSA reduction of ≥ 50% was observed in 24 of 30 patients (80.0%), while a reduction of ≥ 90% was achieved in 14 of 30 patients (46.7%).

## Discussion

In this retrospective analysis of 65 patients, we present, to the best of our knowledge, the first study to investigate the efficacy of [^89^Zr]Zr-PSMA PET/CT in patients with BCR at PSA levels ≤ 0.2 ng/mL.

The key finding was a notably high overall positivity rate of 72.3%. In comparison, standard PSMA PET/CT using short-lived [^68^Ga]Ga-PSMA-11, [^68^Ga]Ga-PSMA I&T, or [^18^F]PSMA-1007 achieved an overall positivity rate of 29.6% in patients with PSA levels ≤ 0.2 ng/mL, as reported in a multicenter study of 321 patients [16]. Depending on the tracer used, positivity rates ranged from 22.5% to 32.4%. Across the literature, particularly for the most established tracer [^68^Ga]Ga-PSMA-11, positivity rates appear highly heterogeneous; nonetheless, the vast majority are markedly lower than the rate observed in the present study for [^89^Zr]Zr-PSMA. Meredith et al. reported a substantially lower positivity rate of 11.3% in patients with PSA levels between 0.01 and 0.2 ng/mL [15], whereas Burgard et al. observed a positivity rate of 25.2% in a previous analysis [16]; in contrast, Hope et al. reported in a meta analysis a higher positivity rate of 58.3% [13]. Afshar-Oromieh et al. investigated the largest BCR cohort reported to date, including 2533 men, and reported a positivity rate of 43.0% in the subgroup of 226 patients with PSA levels ≤ 0.2 ng/mL [14]. Similarly, Hoffmann et al. observed a comparable positivity rate of 41.0% using [^68^Ga]Ga-PSMA-11 in a subgroup of 16 patients with PSA levels < 0.2 ng/mL [28]. Notably, Wang et al. reported a relatively high positivity rate with 73.8% using total-body PET/CT and 43.8% using conventional field-of-view PET/CT for [^68^Ga]Ga-PSMA-11 in patients with PSA levels < 0.2 ng/mL [29]. Overall, detection rates reported for standard PSMA PET/CT with short-lived tracers remain markedly lower than the one observed for [^89^Zr]Zr-PSMA PET/CT in this analysis. In the present study, no statistically significant differences in positivity rates were observed according to PSA level or PSA-DT. This lack of significance may be attributable to the small subgroup sizes. However, there appeared to be a potential trend toward lower positivity rates among patients with very low PSA levels (< 0.1 ng/mL) and longer PSA-DT (> 6 months). In contrast, a statistically significant association between Gleason score and positivity rate was observed, with scores ≥ 8 being linked to higher positivity rates. This suggests that patients with biologically more aggressive BCR may derive particular benefit from this novel imaging approach. The association between higher Gleason score and increased detectability aligns with prior reports from conventional PSMA PET/CT imaging [16, 30]. However, further studies analyzing larger patient cohorts are warranted to confirm and expand these findings on predictive biomarkers.

The superior positivity rate of [^89^Zr]Zr-PSMA PET/CT can be explained by several factors. First, in the setting of PSA levels ≤ 0.2 ng/mL, where very small tumor lesions are to be expected, substantial tracer internalization may require more time than is afforded by the imaging window of conventional radionuclides (half-life of ^89^Zr: 78.4 h vs. 67.7 min for ^68^Ga) [23]. Tumor lesion uptake was reported to increase twofold within the first 24 h and to remain largely constant thereafter [26]. Second, and of particular importance, the half-life of ^89^Zr allows imaging even beyond 24 h after injection (48 h in this study), enabling enhanced tracer clearance from normal organs and background and resulting in markedly improved lesion contrast compared with short-lived standard tracers [26, 30]. Previous analyses have shown that this strategy can increase tumor-to-background ratio by up to 100-fold [26]. Using this approach, small tumor manifestations that often remain concealed on conventional PSMA PET/CT can be visualized with [^89^Zr]Zr-PSMA PET/CT. In a previous study, we observed that in patients with BCR and a previously negative [^68^Ga]Ga-PSMA-11 PET/CT, the positivity rate of [^89^Zr]Zr-PSMA PET/CT was 78.0% [27]. Additionally, the implementation of a late imaging strategy improves the specificity of lesion characterization, enabling a more reliable separation of malignant from nonspecific findings [25].

Early and precise localization of BCR by [^89^Zr]Zr-PSMA PET/CT facilitates individualized, patient-oriented treatment strategies, frequently with curative intent, such as targeted radiotherapy of local recurrence or metastatic lesions within a metastasis-directed therapy framework [31–33]. This is further substantiated by the performed PSA follow-up analysis after [^89^Zr]Zr-PSMA PET/CT-guided targeted radiotherapy, which demonstrated a pronounced PSA decline in the vast majority of patients, underscoring that the irradiated lesions represented true sites of tumor disease. In contrast, in the absence of lesion localization on PSMA PET/CT, radiotherapy is commonly administered empirically to the prostatic bed, which carries an increased risk of misdirected or insufficient treatment. This is of particular relevance in high-grade prostate cancer, where early radiotherapy at very low PSA levels has been associated with superior prognosis [34, 35]. A recent meta-analysis encompassing 34 studies demonstrated that even conventional PSMA PET/CT influences therapeutic management in approximately half of patients with BCR and exerts a substantial impact on radiotherapy planning, including adjustments of target volumes and dose distribution [36]. Moreover, multiple retrospective comparative studies indicate improved clinical outcomes following PSMA-guided radiotherapy [37–40]. Considering the higher positivity rates observed, this impact may be even more pronounced with [^89^Zr]Zr-PSMA PET/CT, which needs to be investigated in future studies.

Although the clinical results are highly promising, the increased patient effort, the logistical challenges related to multiple appointments, and the radiation exposure should not be disregarded. Initial dosimetry studies indicate an exposure of approximately 10 mSv, which is roughly two- to threefold higher than that of short-lived PSMA tracers [17]. However, the logistical constraints and the increased radiation dose appear to be outweighed overall by the clinical benefits.

The limitations of this study include its retrospective, observational nature and single-center design. These characteristics, together with the limited size of cohort and subgroups limit the strength of evidence and generalizability of the findings. Due to the small cohort size, the statistical analysis was limited to univariate and restricted multivariable models; more comprehensive multivariable analyses should be performed in future studies with larger cohorts. It should be acknowledged that the present analysis addresses a biologically heterogenous disease entity, reflecting the well-recognized interindividual variability in PSMA expression. This biological variability is mirrored by the broad range of uptake values and corresponding standard deviations, particularly among patients with PSA levels ≤ 0.2 ng/mL. At the same time, this spectrum of expression highlights the sensitivity of the imaging approach across different biological presentations.

## Conclusion

In patients with BCR and PSA levels ≤ 0.2 ng/mL, [^89^Zr]Zr-PSMA PET/CT yielded a 72.3% positivity rate in this single-center cohort of 65 individuals. This notable sensitivity at minimal PSA concentrations may enable early identification of recurrent disease and facilitate individualized targeted therapy. Future studies, ideally conducted in a prospective setting, are warranted to further evaluate these promising findings.

## Data Availability

The datasets generated during and/or analyzed during the current study are available from the corresponding author on reasonable request.
